# The Independent Evolution Method Is Not a Viable Phylogenetic Comparative Method

**DOI:** 10.1371/journal.pone.0144147

**Published:** 2015-12-18

**Authors:** Randi H. Griffin, Gabriel S. Yapuncich

**Affiliations:** Department of Evolutionary Anthropology, Duke University, Durham, North Carolina, United States of America; Scientific Research Centre, Slovenian Academy of Sciences and Arts, SLOVENIA

## Abstract

Phylogenetic comparative methods (PCMs) use data on species traits and phylogenetic relationships to shed light on evolutionary questions. Recently, Smaers and Vinicius suggested a new PCM, Independent Evolution (IE), which purportedly employs a novel model of evolution based on Felsenstein’s Adaptive Peak Model. The authors found that IE improves upon previous PCMs by producing more accurate estimates of ancestral states, as well as separate estimates of evolutionary rates for each branch of a phylogenetic tree. Here, we document substantial theoretical and computational issues with IE. When data are simulated under a simple Brownian motion model of evolution, IE produces severely biased estimates of ancestral states and changes along individual branches. We show that these branch-specific changes are essentially ancestor-descendant or “directional” contrasts, and draw parallels between IE and previous PCMs such as “minimum evolution”. Additionally, while comparisons of branch-specific changes between variables have been interpreted as reflecting the relative strength of selection on those traits, we demonstrate through simulations that regressing IE estimated branch-specific changes against one another gives a biased estimate of the scaling relationship between these variables, and provides no advantages or insights beyond established PCMs such as phylogenetically independent contrasts. In light of our findings, we discuss the results of previous papers that employed IE. We conclude that Independent Evolution is not a viable PCM, and should not be used in comparative analyses.

## Introduction

Phylogenetic comparative methods (PCMs) provide a wide array of analytical tools for investigating evolutionary questions given a phylogeny and trait values at the tips of the tree. Some of the most common PCMs include methods for estimating ancestral states, investigating correlated evolution among multiple traits, testing for different rates of evolution in different parts of a phylogeny, and comparing evolutionary models (reviewed in [1–3]). As the set of available methods continues to grow in number and sophistication, evolutionary biologists must decide which among these PCMs is most appropriate for addressing their research questions. It is critical for researchers to ensure that the methods they employ are theoretically sound and validated by relevant simulation studies.

In 2009, Smaers and Vinicius [4] proposed a new PCM, Independent Evolution (IE), which estimates ancestral states and separate rates of evolution along each branch of a phylogeny. The IE method purportedly incorporates the assumptions of an “adaptive peak” model of evolution [5], in which a population attempts to reach a wandering adaptive peak. Smaers and Vinicius [4] state that their method produces equivalent results to simpler models of evolution, such as Brownian Motion (BM) and Ornstein-Uhlenbeck (OU), when the assumptions of those models are met. Additionally, when branch-specific rates of evolution are estimated independently for a pair of traits (e.g., brain size and body size) and plotted against one another, Smaers et al. [6] proposed that deviations from the line *y* = *x* indicate deviations from the taxon-specific allometric relationship on specific branches of the tree, since “the allometric slope of the brain-body relationship collapses into the isometric line when plotting rates of change” ([6], p. 18008). These deviations are then interpreted as indicating selection on one or both of the traits under investigation (see Fig 3 in [6]). Since its introduction, the IE method has been used to study the correlated evolution of particular brain structures in primates [7–9], brain and body size in several mammalian orders [6], trabecular bone structure and wrist morphology in hominoids [10,11], levels of phenotypic integration in the crania of carnivorans [12], and rates of cranial evolution within Carnivora [13].

Despite its increasing popularity, the IE method has not been subject to a rigorous investigation of its statistical performance. Although Smaers and Vinicius [4] performed simulations to compare IE to several existing methods, their simulations are inadequate for three reasons. First, it appears that the simulated data were not treated appropriately for alternative PCMs. Specifically, raw values were used rather than logarithmically transformed values. As the error structures of many biological variables display geometric normality (in which deviations from the mean differ by equal proportions) rather than arithmetic normality (in which deviations differ by equal amounts) [14], data transformation is often required to reduce heteroscedasticity and remove the positive correlation between a trait’s mean value and its variance [15]. The alternative PCMs compared by Smaers and Vinicius [4] all require trait means and their variance to be uncorrelated; this is most frequently achieved by logarithmic transformation [1,16–19]. Since the IE algorithm incorporates a step that accounts for trait proportionality, the authors do not transform their simulated (and geometrically normal) data prior to analysis. However, the lack of log-transformation of the simulated data likely reduced the effectiveness of alternative PCMs in their simulations. Second, Smaers and Vinicius’ [4] simulation study only reports results for the performance of IE in ancestral state reconstruction for a few nodes in the phylogeny, but the most frequent application of the IE method has been to estimate rates of evolution for all branches in a phylogeny [6–11,13], and they did not assess the accuracy of IE in this context. Finally, no simulations were done to assess the performance of the method in the context of comparing rates of evolution between pairs of traits, but this has also been a frequent application of the method [6–9].

At the onset of our study, our goals were twofold: to reassess IE’s ability to reconstruct ancestral state accurately when geometrically normal datasets are log-transformed prior to analysis with alternative PCMs that require it, and to test IE’s ability to recover accurate rates of evolution on individual branches of the tree. However, in the course of our study, we discovered substantial theoretical and computational issues with the IE method. As a result, this report now has four major objectives: 1) to highlight the problems with the IE method; 2) to use computer simulations to evaluate the accuracy of IE ancestral state reconstructions and estimation of branch-specific rates of change; 3) to use computer simulations to assess the claim that regressing branch-specific changes for one trait against another yields information beyond that gleaned from a traditional phylogenetic regression; and 4) to reinterpret results from past studies that use IE.

### The Independent Evolution method

**Fig 1:**
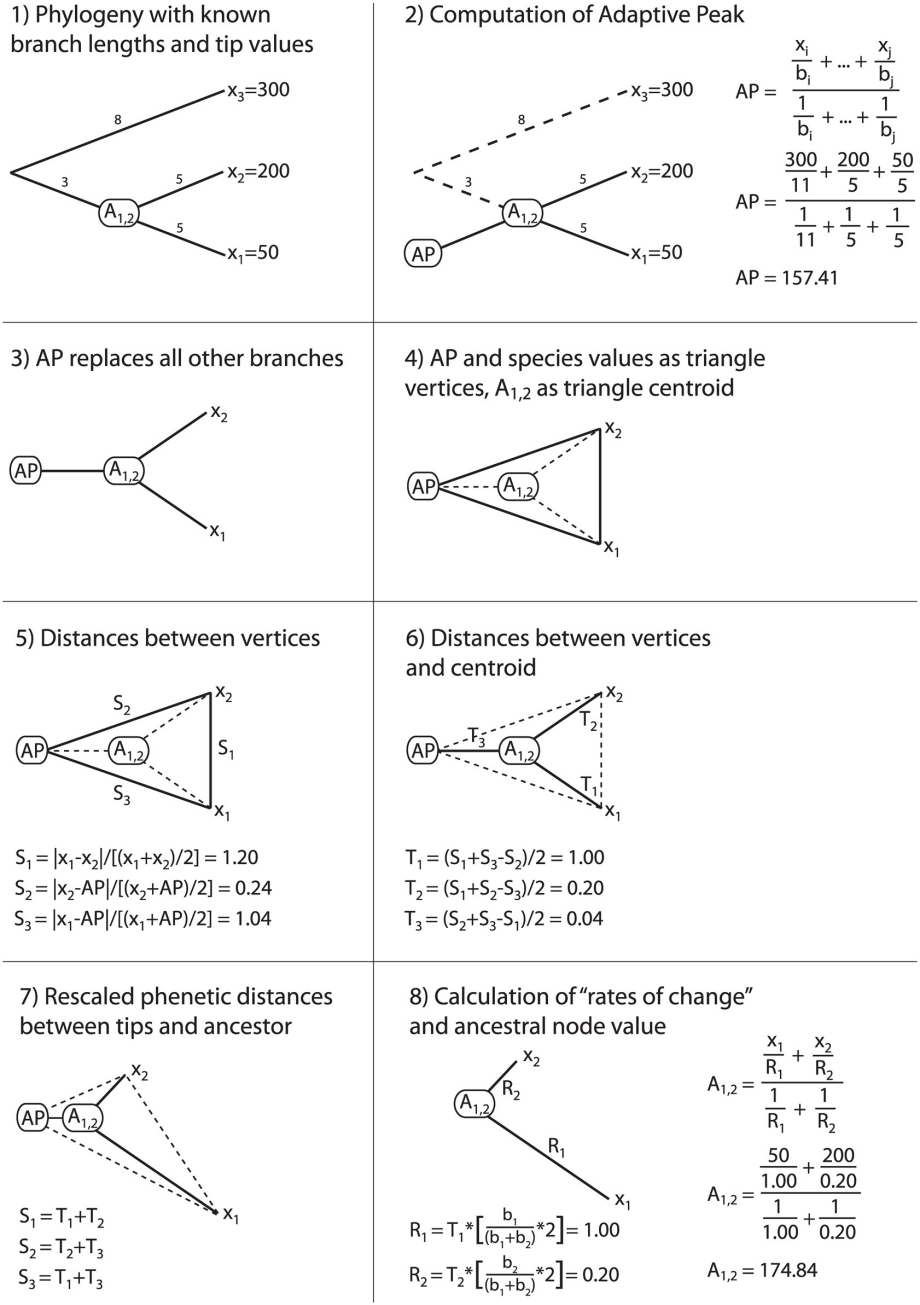
The Independent Evolution method. Steps for the algorithm are detailed in the main text. Modified from Smaers and Vinicius [4].

The IE method [4] is an algorithm for traversing a phylogeny and reconstructing ancestral states for each internal node. “Rates of change” are then calculated between ancestral and descendant nodes for every branch in the phylogeny. Smaers and Vinicius [4] provide an eight-step algorithm along with an example, illustrated in Fig 1. The algorithm is as follows:

1. Given a fully resolved phylogeny with known branch lengths and observed trait values for all terminal taxa, an “adaptive peak” value (*AP*) is estimated for the ancestral node (*A*_1,2_) for two sister taxa, *x*_1_ and *x*_2_ (Fig 1.1).
2. *AP* is calculated by summing the tip values of all terminal taxa weighted by their distances along branches to *A*_1,2_; this sum is then divided by the sum of the inverse distances along branches to *A*_1,2_ for all terminal taxa (Fig 1.2). This step extends Felsenstein’s equation for computing the ancestral state of two sister nodes (Equation 3 in [20]) to the entire tree. Note that the error structure of the data is not examined and the data are not transformed prior to this step.
3. The branch connecting *AP* to *A*_1,2_ replaces all other branches in the phylogeny, creating a star phylogeny linking *AP*, *A*_1,2_, *x*_1_, and *x*_2_ (Fig 1.3).
4. *AP*, *x*_1_, and *x*_2_ are then considered vertices of a triangle with *A*_1,2_ as the centroid (Fig 1.4).
5. Side lengths (*S*_i_) of the triangle are calculated with the IE distance metric: the absolute value of the difference between two vertices divided by their average (Fig 1.5). The authors claim that the IE distance metric has “equivalent properties to the log-scale” ([6], p. 18010).
6. Distances from each vertex to the centroid (*T*_i_) are calculated using formulas developed by Farris [21,22] as the sum of two sides minus the third, divided by two (Fig 1.6). Smaers and Vinicius [4] justify this step by appealing to Ptolemy’s triangle inequality theorem, which states that one side of a triangle will be always be less than or equal to the sum of the other two sides [23].
7. Smaers and Vinicius [4] then claim that the distances from each vertex to the centroid (*T*_i_) represent “the relative phenetic distances” (pg. 995) between ancestor and descendants when the adaptive peak is considered (Fig 1.7).
8. An *R*-value representing the “relative branch-specific evolutionary change” (*R*_i_) for each descendant branch is calculated by multiplying the *T*-distance by a scaled branch length (e.g., branch length *b*_1_ divided by the sum of branch lengths *b*_1_ and *b*_2_, multiplied by two). Finally, the ancestral node value is calculated as the average of the trait values for the sister taxa *x*_1_ and *x*_2_, weighted by the *R*-values (Fig 1.8). This step only returns positive *R-*values, so a *post hoc* procedure may be applied in which the *R*-values of branches with a decrease in the trait value are multiplied by –1.

When the IE algorithm is applied to find all ancestral states and *R-*values in the tree, adaptive peaks are first calculated for all internal nodes, and then the tree is traversed from the tips to the root in order to compute ancestral states. During this tree traversal, descendant branches are collapsed once the ancestral state is computed for their parent node, and the computed ancestral state is incorporated into ancestral states of nodes deeper in the tree. In the illustrated example, *x*_3_ and *A*_1,2_ would represent triangle vertices and branch lengths of 8 and 3 would be used to find the value of the root node.

### Problems with the Independent Evolution method

We have identified multiple theoretical and computational problems with the IE method. We describe each of these in detail below, and briefly list them here: 1) the IE distance metric is intended to account for relative differences, but introduces bias and generates undefined values; 2) the IE distance metric makes the implicit assumption that observed trait values and their variance are positively correlated and thus require transformation, but this positive correlation does not hold for all data types; 3) the authors have misapplied Farris’ [21,22] equations to calculate *T*-distances; 4) *R*-values are described as “rates of change” along individual branches of the phylogeny, but they are not truly evolutionary rates; 5) geometrically normal traits are not transformed prior to calculating adaptive peak values, such that large values have an disproportionate impact on adaptive peaks and ancestral states throughout the tree; 6) the IE algorithm estimates 2*n* — 2 “rates of change” from *n* observations, such that these estimates necessarily contain redundant information; 7) the justification for extending Felsenstein’s [20] equation to the entire tree in order to calculate adaptive peaks is suspect; and finally, 8) when branch-specific changes for one trait are regressed against those of a second trait, the results do not provide any additional information which could not be gleaned from a standard phylogenetic regression of those two traits.

#### 1. The IE distance metric

In order to account for proportional rather than absolute differences in trait change, Smaers and Vinicius [4] utilize the following distance metric:

$$S=2\times \left(\frac{\left|x-y\right|}{x+y}\right)$$ (1)

where *x* and *y* represent trait values in arithmetic space, and *S* is the proportional distance between *x* and *y*. The IE distance metric is identical to an asymmetry index frequently applied to linear measures of the brain [24,25] or skeleton [26–28]. The IE distance metric is also identical to Storer’s index, a measure of sexual size dimorphism used primarily in ornithology [29–32].

**Fig 2:**
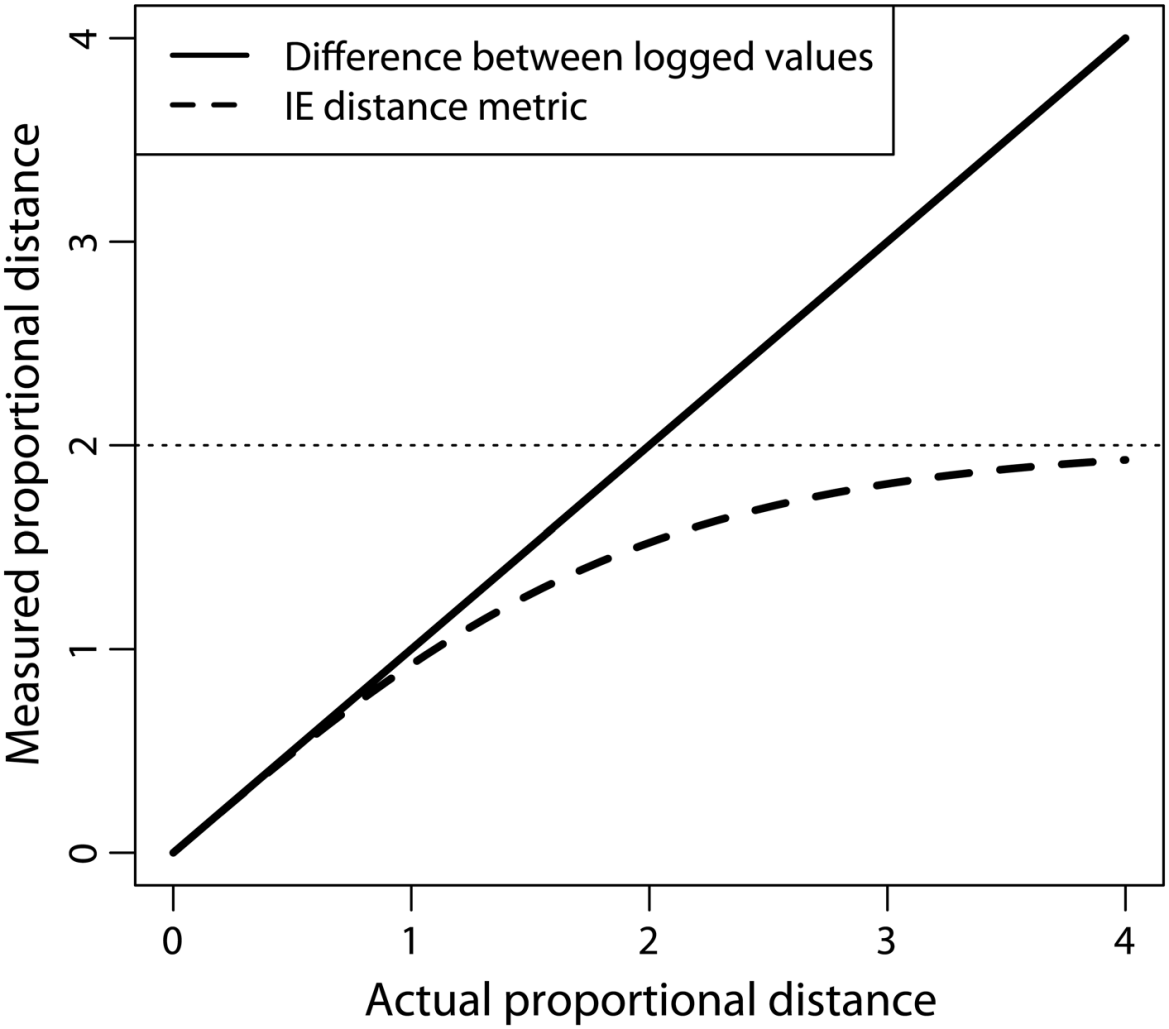
Comparison of distance metrics. The IE distance metric asymptotes at |2|, leading to severely underestimated distances when the difference between values is large. In contrast, the difference between logged values does not asymptote, and is an unbiased estimator of proportional distances.

Smaers et al. [6] claim the IE distance metric has “equivalent properties to the log-scale” (p. 18010), but this is decidedly not true. Smith [33] has emphasized that this formula is *not* linear: as the difference in values increases, the IE distance metric asymptotes at ±2. Therefore, Smith [33] recommended that the difference between logged values be used as an unbiased estimator (ln[*x*] − ln[*y*], which is equivalent to ln[*x*/*y*]). Though bias introduced by the IE distance metric is less extreme when the difference between values is small (as in asymmetry studies), strong bias can be generated when examining potentially large differences such as the difference in body mass between two species. The IE distance metric will always underestimate proportional change, and the magnitude of this underestimation will be greater when the difference between trait values is large (Fig 2).

**Fig 3:**
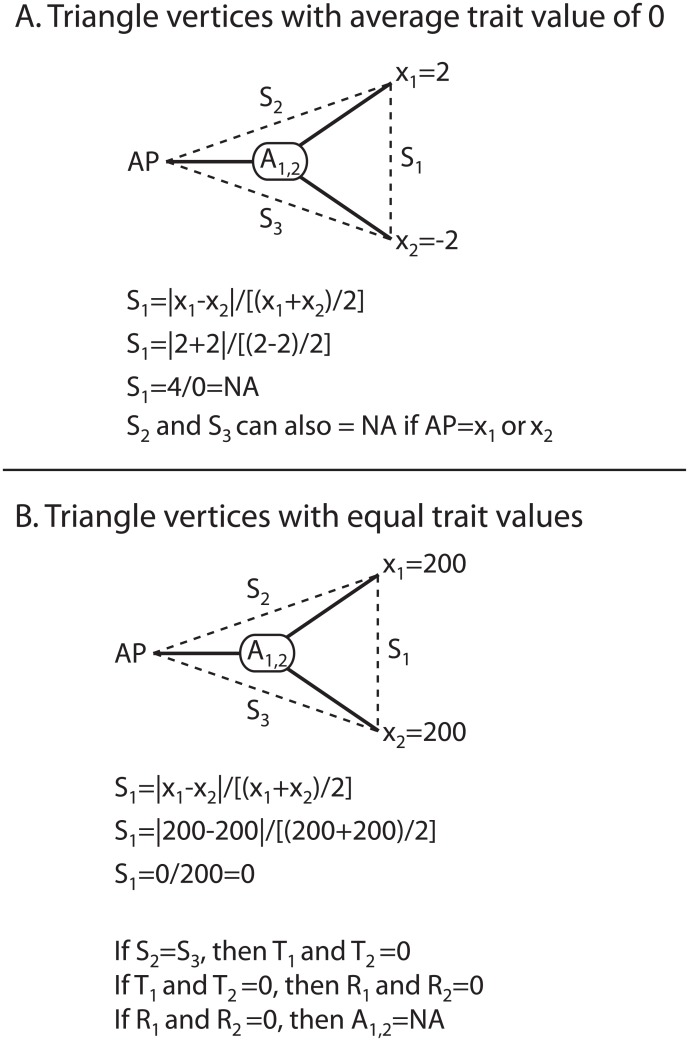
Computational problems with the IE method. A) When the average of triangle vertices (some combination of *x*_1_, *x*_2_, and/or *AP*) is zero, side lengths will be undefined. B) When triangle vertices have equal values, the ancestral state value will be undefined.

In addition to distorting distances, the IE distance metric introduces several computational problems (Fig 3). First, the distance metric will be undefined if the average of any two vertices (either tip values or the adaptive peak), and thus the denominator of the equation, is equal to 0 (Fig 3a). This can occur if the traits being analyzed include negative values, as in the case of using principal component scores (e.g., [13,15]). Second, the ancestral value will be undefined if any two vertices have the same value (Fig 3b), as one triangle side will be equal to zero, and therefore the *T*-distances from *A*_1,2_ to *x*_1_ and *x*_2_ will be equal to zero. If *T*_1_ or *T*_2_ is equal to zero, then the corresponding *R*-value is equal to zero, and the ancestral node value will be undefined since *R*-values are in the denominator of the ancestral node value.

#### 2. Embedding data transformation into the IE algorithm

The IE distance metric transforms distances between ancestors and descendants in order to account for proportional rather than absolute changes. As a data transformation step, the IE distance metric makes the assumption that there is a positive correlation between trait means and their variance for *all* data types (i.e., that all traits exhibit geometric normality). While this assumption is valid for many morphological variables (e.g., lengths, areas, and volumes of structures), there are also many data types that exhibit arithmetic normality (e.g., angular measures, principal component scores). In these cases, transforming distances with the IE distance metric is likely to generate a negative correlation between means and variances, such that distances between large values are inappropriately reduced. Since the IE method includes a data transformation step, the algorithm should only be applied to data that exhibits geometric normality. However, the authors do not highlight this assumption, and the IE algorithm has been applied to data that violate it (e.g., principal component scores in [11] and [13]).

#### 3. Misapplication of Wagner tree formulas and the Ptolemean triangle inequality theorem

Smaers and Vinicius [4] apply formulas developed by Farris [21,22] to calculate the distances from triangle vertices (*x*_1_, *x*_2_, *AP*) to the centroid (*A*_1,2_) or barycentre of the triangle (Fig 1.6). Farris’ [21,22] formulas detail the process by which a Wagner tree (a minimum length tree representing a parsimonious solution to connecting operational taxonomic units [OTUs]) can be generated using the distance matrix of those OTUs. Generating a minimum length tree from a distance matrix is not the same problem as determining distances within a known phylogenetic tree. These formulas were not intended for use in a geometric context and cannot be used to compute the distances from triangle vertices to the centroid. The correct distances from triangle vertices to the centroid can be obtained by using Apollonius’ theorem to compute the lengths of the triangle medians (i.e., line segments connecting each triangle vertex to the midpoint of the opposite side), and then using the triangle property that the distance between a vertex and the centroid is 2/3 the length of the median connected to that vertex. By Apollonius’ theorem, we have:

$$m_{a}\;=\;\sqrt{\frac{2b^{2}+2c^{2}+a^{2}}{4}}$$ (2)

where *a*, *b*, and *c* are the triangle sides, and *m*_*a*_ is the median of side *a*. If vertex A is the vertex opposite side *a*, then the distance from vertex A to the centroid is given by:

$$T_{A}=\frac{\sqrt{2b^{2}+2c^{2}-a^{2}}}{3}$$ (3)

Applying this approach to the triangle in Fig 1.6 with side lengths *S*_1_ = 1.2, *S*_2_ = 0.24, and *S*_3_ = 1.04, we find that the true *T*-distances should be *T*_1_ = 0.74, *T*_2_ = 0.46, and *T*_3_ = 0.31. These values are quite different from those computed in Fig 1.6, revealing that Smaers and Vinicius’ [4] application of Wagner tree formulas does not accurately measure the distances from triangle vertices to the centroid.

Smaers and Vinicius [4] then cite the Ptolemean triangle inequality theorem as justification for the formulas they utilize in Step 7 of the IE algorithm. The Ptolemean triangle inequality theorem states that one side of a triangle will be always be less than or equal to the sum of the other two sides. When one side is equal to the sum of the other two sides, the triangle has an area of zero and can be represented by a line [23]. In Step 7 of the IE algorithm, the authors claim that each *S*-distance will be equal to the sum of two *T*-distances (Fig 1.7). However, by the very theorem they cite, if *S*_1_, *T*_1_, and *T*_3_ are sides of a triangle and *S*_1_ = *T*_1_ + *T*_3_, then the triangle formed by *S*_1_, *T*_1_, and *T*_3_ has an area of zero. In fact, all triangles formed by two *T*-distances and an *S*-distance have areas of zero and can be represented solely by *S*-distances. Taken together, the equations presented in Steps 6 and 7 of the IE algorithm are nonsensical.

#### 4. R-values are not evolutionary rates

In Step 8 of the IE algorithm, *T*_1_ and *T*_2_ are weighted by their respective branch lengths to produce “*R*-values”, which Smaers and Vinicius [4] refer to as “branch-specific rates of evolutionary change”. However, if rates are defined as a distance divided by time, these quantities should not be considered rates. As we have shown, the *S-*distances and *T*-distances upon which the *R*-values are based are problematic due to the biased IE distance metric used to compute *S*-distances and the inappropriate use of Wagner’s formulas to compute *T*-distances. Given these foundational issues, it is difficult to say what the *R*-values may represent in biological terms. But even if the *T*-distances represented “phenetic distances” from the ancestral state to the descendant states as the authors claim, the equation used to weight the *T*-distances by branch lengths would not result in a rate. Given phenetic distance *T* between an ancestor and descendant, and branch length *b* connecting them, the average rate of change along the branch could be computed as *T/b*. In comparison, Step 8 of the IE algorithm multiplies *T*-distances by the quantity 2*b_1_/ (*b*_1_ + *b*_2_), where *b*_1_ and *b*_2_ are branch lengths leading from the ancestral node to sister descendants. It is unclear why this particular equation is used to scale *T*-values, but we see no justification for interpreting the resulting *R*-value as a rate of evolutionary change.

The IE method differs notably from other PCM approaches to conceptualizing and estimating evolutionary rates for comparative data. The most common approach for studying evolutionary rates involves modeling evolution as a statistical process, most frequently a constant-variance Brownian motion process, where changes along individual branches are drawn from a normal distribution with a mean of 0 and the variance proportional to branch lengths. This approach treats changes along individual branches as random variables and provides a framework for assessing whether rates of evolution differ significantly across different branches or parts of the tree. In contrast, the IE method treats changes along individual branches as opportunities to estimate the rate of evolution separately for each branch of the phylogeny, which does not allow for the quantification of uncertainty and risks treating random noise as meaningful variation in evolutionary rates.

#### 5. The adaptive peak’s “pull” towards extreme values

The IE algorithm is applied to untransformed geometrically normal data, but it does not account for the proportionality of traits when computing the adaptive peak. While the IE distance metric provides a partial (though biased) correction for proportionality, this metric is not applied until after the adaptive peak has been computed from raw trait data (Fig 1.2). Thus, extreme tip values have a strong impact on adaptive peak values throughout the tree, and the IE algorithm mis-estimates the weighted average of the tips. Since ancestral states are triangulated from these adaptive peaks, the IE algorithm will systematically bias ancestral states. The IE algorithm has primarily been applied to traits with positive values (such as body or brain mass); in these cases, the bias is expressed by a systematic overestimation of ancestral states.

#### 6. Statistical non-independence of ancestral states and branch-specific rates of change

IE aims to estimate branch-specific rates of change for every ancestor-descendant pair in the phylogeny, such that the algorithm generates 2*n –* 2 estimates from *n* observations. Unfortunately, these estimates cannot be statistically independent of one another because some ancestors are also descendants, and are therefore components of multiple estimates. Non-independence is also a major problem for estimated adaptive peaks and ancestral states, since the same set of tip values are used to compute all *n–* 1 adaptive peaks, and the adaptive peaks are then used to triangulate ancestral states throughout the tree. Thus, the IE algorithm fails to address a central issue that PCMs seek to alleviate: the statistical non-independence of species data.

#### 7. The “Adaptive Peak” model

There is no convincing theoretical reason for extending Felsenstein’s [20] equation for computing ancestral states to the entire phylogeny and calling it an “adaptive peak”. The theoretical weakness of this approach can be highlighted with a thought experiment. As discussed earlier, the IE algorithm is intended to be applied to untransformed geometrically normal data, but the asymptotic IE distance metric does not adequately correct for this (Fig 2). We can ask the question: what happens if we apply the IE algorithm to log-transformed data and replace the IE distance metric with the difference between logged values as an appropriate measure of proportional distance [33]?

**Fig 4:**
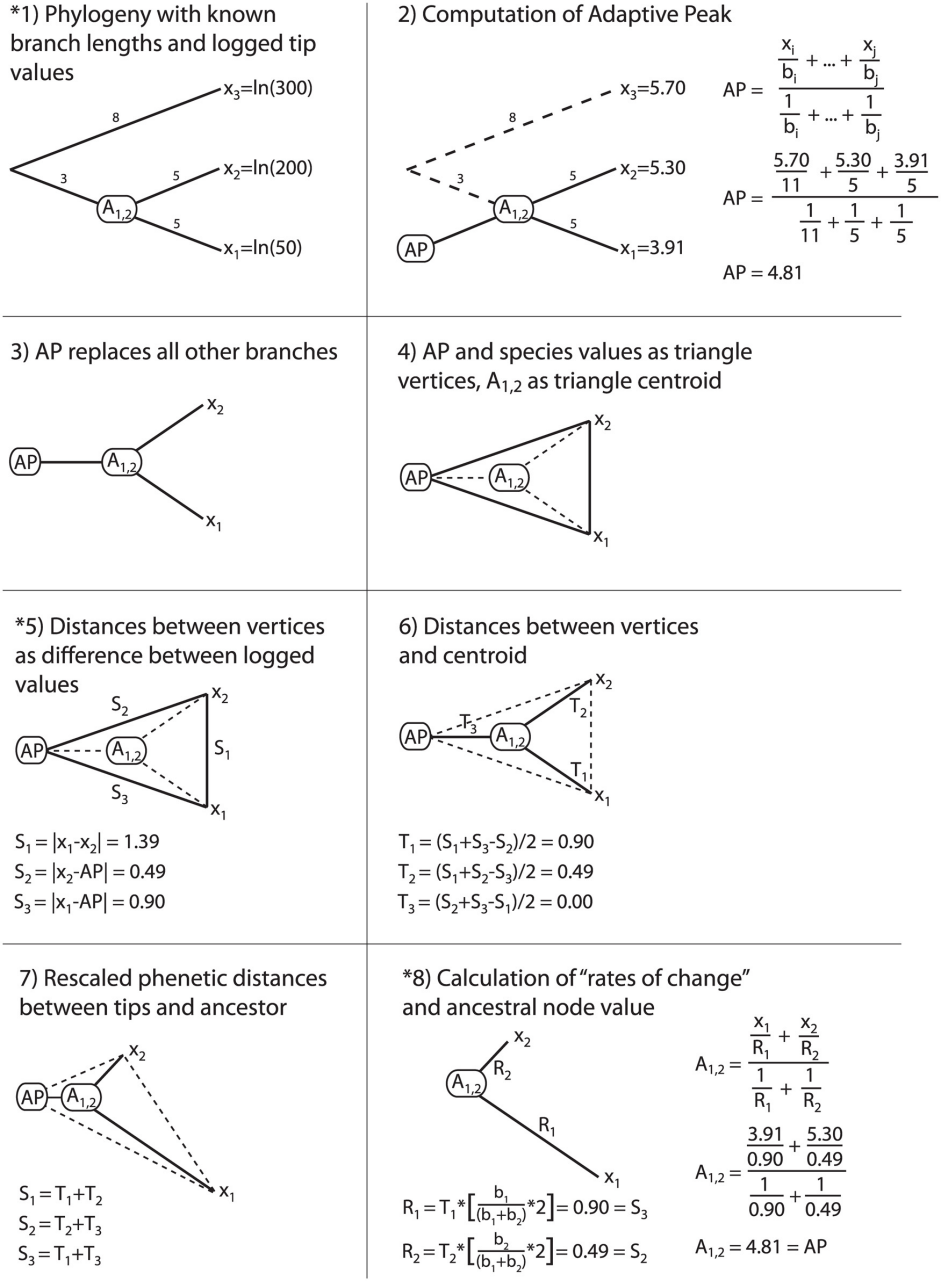
Modified Independent Evolution algorithm. Asterisks indicate steps that have been modified. In Step 1, data are log-transformed prior to analysis so that extreme values do not cause the overestimation of adaptive peaks. In Step 5, the difference between logged values replaces the IE distance metric, so that proportional distances are not underestimated. All other steps of the algorithm remain the same. With these two modifications, the adaptive peak computed in Step 2 is equivalent to the ancestral state value in Step 8.

Fig 4 shows the outcome of this experiment when applied to the original example provided by Smaers and Vinicius [4]. With the new (unbiased) distance metric, distances from extant species to the adaptive peak (*S*_2_ and *S*_3_) are equal to the distances from extant species to the ancestral node (*T*_2_ and *T*_1_), and the distance from the adaptive peak to the ancestral node (*T*_3_) is 0 (Fig 4.6). This suggests that the ancestral node value will have the same value as the adaptive peak. Working through the example confirms that the ancestral state is now identical to the adaptive peak (Fig 4.8). In Appendix 1, we provide an algebraic proof to demonstrate that whenever the branch lengths leading to the sister taxa are equal and the adaptive peak value is between *x*_1_ and *x*_2_, the adaptive peak will be equal to the ancestral state (Fig 4.8). The correspondence of the adaptive peak and ancestral state shows the recovery of an “adaptive peak” in Fig 1 is a byproduct of the failure to transform geometrically normal data prior to computing the adaptive peak value, and distortion introduced by the IE distance metric. When the correlation between trait values and variance is removed at the beginning of the algorithm and an appropriate metric is used to compute distances between trait values, the “adaptive peak” disappears. Of course, branch lengths will not necessarily be equal for ancestral state reconstructions involving internal nodes, so the equivalence of the ancestral state and adaptive peak is only expected for the ancestors of pairs of extant sister species. However, there is no theoretical reason why the common ancestors of extant sister taxa have always reached adaptive peaks, while the common ancestors of nodes deeper in the tree have not.

Smaers and Vinicius [4] provide only a vague justification for their approach. They state that a primary benefit of their approach is that their equation for computing adaptive peaks “takes into account all available (i.e., extant) biological information provided…to increase the reliability of character optimization” (p. 997). However, as optimization involves selecting the best value from a set of options according to some criterion (e.g., likelihood), and as the IE method lacks any optimization criterion, this claim misrepresents what the algorithm does. Moreover, their claim implies that other PCMs fail to take into account all available biological information, which is not true; most modern methods of ancestral state reconstruction are based on likelihood and utilize all available biological information when estimating parameters [34]. Finally, Smaers and Vinicius [4] suggest that the IE method provides a more general model than common statistical models of evolution such as Brownian Motion (BM) or Ornstein-Uhlenbeck (OU), arguing that IE “allows the inclusion of specific models of evolution such as BM and OU as special cases” (p. 997). Specifically, they claim the model “collapses into a BM model of evolution when *S*_2_ equals *S*_3_” (p. 997). Unfortunately, this statement is not true under further scrutiny: the only way for *S*_2_ to equal *S*_3_ is for *x*_1_ to equal *x*_2_ (Fig 1.5), and as demonstrated in Fig 3b, the ancestral state is undefined when *x*_1_ = *x*_2_. More fundamentally, unlike BM and OU, IE is not based on an explicit statistical model, making the assertion that the adaptive peak model can “collapse” into BM or OU quite perplexing since it implies that the adaptive peak model is an explicit statistical model in which BM and OU are nested.

#### 8. Use of IE branch-specific “rates of change” in regression analysis

**Fig 5:**
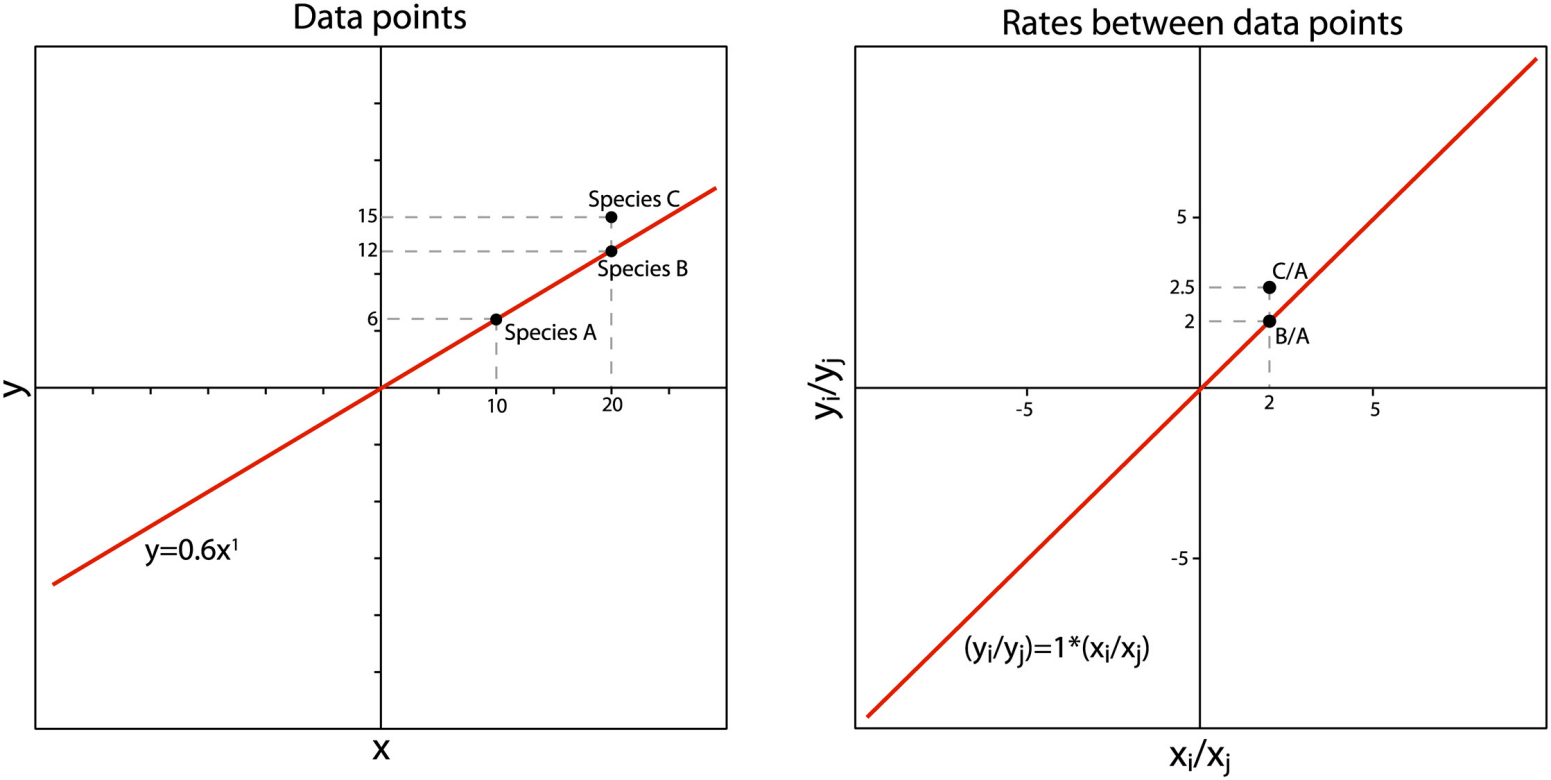
Reproduction of Fig 3 from Smaers et al. [6]. The authors claim that allometry “collapses” into isometry when rates of change between ancestor-descendant species are plotted rather than trait values. In this figure, Species A is ancestral to both Species B and C. From the original figure, it is unclear if trait values have been log-transformed or not.

A common application of the IE method has been to compare “rates of change” along individual branches for a pair of coevolving traits [6–9]. We have already argued that IE does not actually estimate rates of change along individual branches. We have also highlighted that IE estimates of branch-specific change are not statistically independent, which leads to a pseudoreplication problem when they are treated as independent data points in subsequent statistical analyses. However, even if we grant that IE *can* estimate 2*n*—2 statistically independent branch-specific rates of change for a given trait, recent application of IE reveals a serious misinterpretation of the linear regression slope estimated for two sets of IE branch-specific “rates of change” [6]. Smaers et al. [6] argue that the null expectation for this regression slope should be 1, claiming that an allometric scaling relationship will “collapse” into isometry (the line *y* = *x* in a volume ~ volume relationship) when rates of change between data points are plotted rather than the data points themselves (their Fig 3, modified in our Fig 5). They then interpret deviations from the line *y* = *x* as providing novel insights to the evolutionary process leading to observed trait covariation on each branch of the phylogeny.

**Fig 6:**
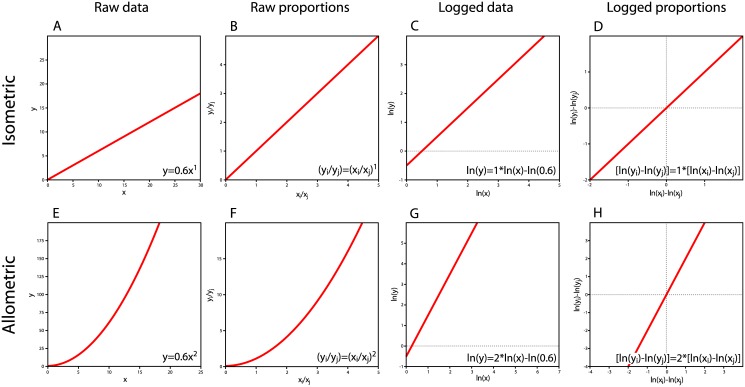
Isometric and allometric scaling relationships for raw values, logged values, and proportions of both types of values. Both *x* and *y* are of the same dimensionality, as in a brain mass ~ body mass relationship. In an isometric relationship, the scaling exponent does not change when raw values in arithmetic space (A) are plotted compared to raw proportions in arithmetic space (B). When data are log-transformed (C), the scaling exponent becomes the slope of the line. The same is true when proportions are plotted in log-space (D). For raw data (E) or raw proportions (F), an allometric relationship can be recognized by a scaling exponent that is not equal to 1 (again assuming equivalent dimensionality of *x* and *y*). For logged data (G) or logged proportions (H), allometry is characterized by a slope not equal to 1. In all iterations, isometric relationships remain isometric and allometric relationships remain allometric. There is no “collapse” of allometry into isometry as suggested by Smaers et al. [6]. The authors either misidentify (A) as an allometric relationship, or do not appropriately convert logged data (C) into logged proportions (D).

This proposal represents a misunderstanding of either allometry or the appropriate treatment of log-transformed data. Smaers et al. [6] do not state whether the data plotted in Fig 5 (their Fig 3) are raw values or if they have been log-transformed. However, the implications of their proposal under either possible treatment are erroneous. Assuming the data are not log-transformed and both axes have the same dimensionality (as in a brain/body mass relationship), then the pictured scaling relationship (*y* = 0.6*x* ^1^) does not represent allometry in arithmetic space. In arithmetic space, allometric relationships are curvilinear, and one variable scales exponentially relative to the other (Fig 6a). When log-transformed, the equation *y* = 0.6*x* ^1^ becomes ln(*y*) = ln(0.6) + 1*ln(*x*) (Fig 6c), which is an isometric relationship (i.e., the scaling coefficient is 1). Thus, assuming the data points are not log-transformed in Fig 5, rather than showing allometry “collapsing” into isometry, Smaers et al. [6] demonstrate that an isometric relationship in arithmetic space “collapses” into an isometric relationship in geometric space.

**Fig 7:**
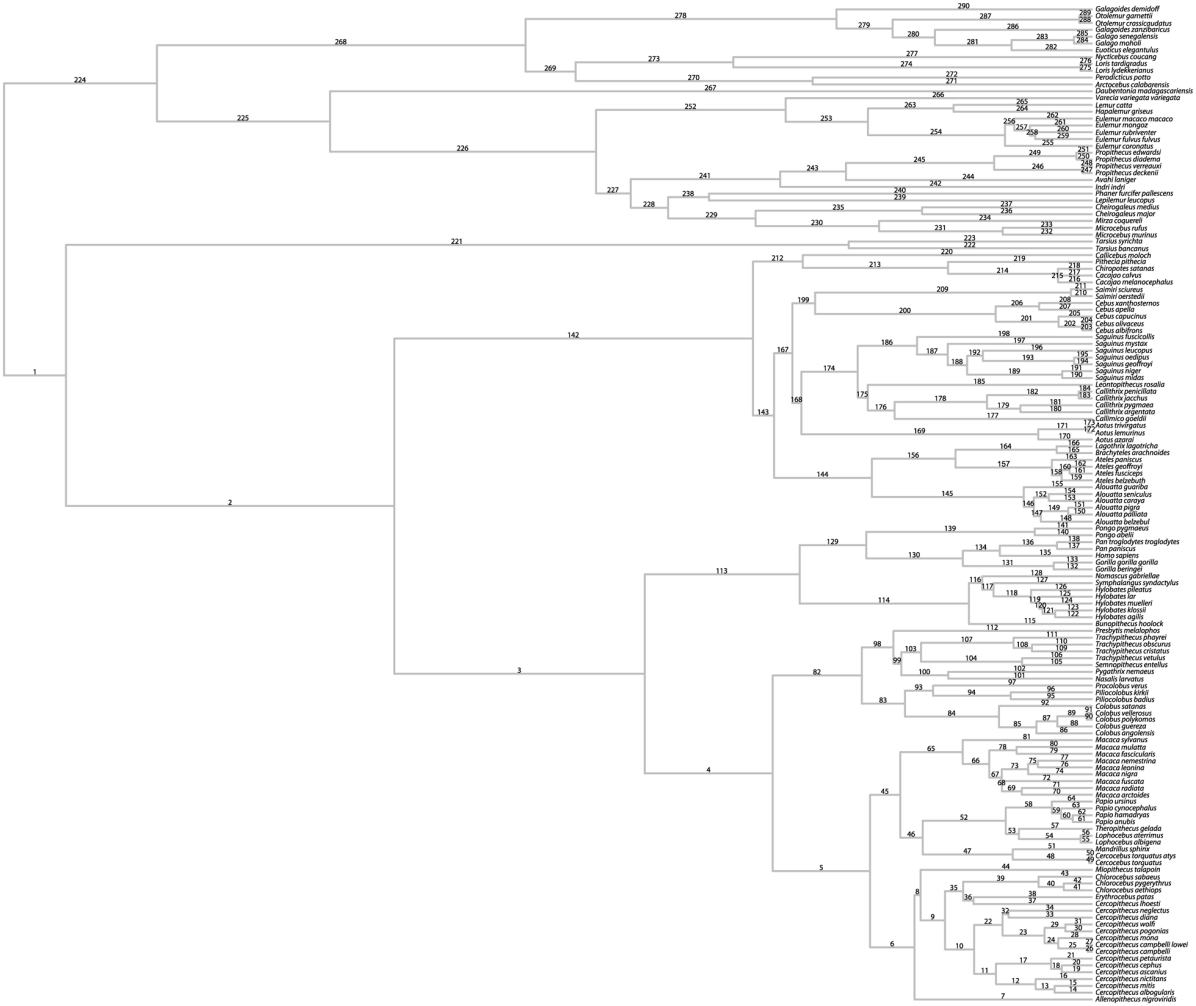
Phylogenetic tree used for simulation studies. Numeric labels on branches correspond to the branch numbers referenced in subsequent figures.

Alternatively, if the data have been log-transformed (Fig 7c and 7g), then proportional changes cannot be calculated with a ratio, as Smaers et al. [6] apparently have done. In log-space, proportional changes must be calculated by taking the difference between log-transformed values as ln(*x*_1_/*x*_2_) = ln(*x*_1_) − ln(*x*_2_) [20,35] (Fig 7d and 7h). For a pair of geometrically normal traits that are perfectly correlated, the scaling coefficient for log-transformed values will be equivalent to the slope of the best-fit line through the differences between log-transformed trait values (Fig 7c, 7d, 7g and 7h). Plotting differences between log-transformed trait values will not change the slope of the best-fit line, but will set the intercept to zero (Fig 7d and 7h).

We have shown that the null expectation for the regression slope estimated for one set of IE branch-specific changes against another is not 1, as argued by Smaers et al. [6]. What, then, is our expectation for the regression slope? Under IE, each data point in the analysis represents IE-inferred evolutionary change for a pair of traits along a single branch, based on comparing the trait values for the ancestor and the descendant. This use of ancestor-descendant contrasts strongly resembles a previous method for detecting correlated evolution known as “minimum evolution” (ME) [35–38], which is part of a class of methods known as “directional contrasts” [38,39]. In most implementations of ME, ancestral node values were estimated with squared-changed parsimony [36–38], directional contrasts were taken between ancestral and descendant nodes, and the contrasts of two traits were regressed against one another in order to examine the correlation of changes [36].

Initial evaluations of directional contrasts [38] suggested they provided different evolutionary insights than “cross-sectional” techniques such as phylogenetically independent contrasts (PIC; [20]), which make use of contrasts between pairs of sister taxa rather than ancestors and descendants. However, the viability of directional contrast methods was severely undermined by Pagel [40,41], who demonstrated that despite differing ways of calculating contrasts, directional and cross-sectional phylogenetic comparative methods both estimate the same parameter of interest, the “evolutionary regression coefficient”. Pagel [40,41] argued that cross-sectional techniques, such as PICs, should be preferred over directional contrasts based on statistical properties: because directional contrasts compute more contrasts (2*n*—2) than the available degrees of freedom (*n*– 1), much of the information contained in the contrasts is redundant, resulting in elevated Type I errors [38]. Following these observations, directional contrasts have fallen out of favor in comparative biology: Huey and Bennett reanalyzed their data with PIC and an updated phylogeny [42], very few researchers have used directional contrasts over the past two decades [43,44], and the method has received little to no attention in reviews of PCMs (e.g., [1–3,45–47]). Garland et al. [45] went so far as to call ME a “partially phylogenetic” method (p. 3027).

With the recognition that the IE method shares many similarities with the ME approach for estimating evolutionary regression coefficients, we reasoned that the regression slope for a pair of IE branch-specific rates of change reflects the evolutionary regression coefficient, or the allometric scaling coefficient, for that pair of traits. In the following section, we use simulations to demonstrate that this is true.

## Methods

### Simulations: evolution of a single trait

All simulations and analyses were performed in R [48]. We simulated the evolution of 1000 geometrically normal traits under a constant-variance BM model of evolution. This was achieved using the fastBM function in the phytools package [49] to simulate BM evolution with mean 0 and variance 1, and then exponentiating the simulated trait values at internal nodes and tips. Thus, in log-space, the expected value of the simulated ancestral states at every node is 0, with gradually increasing variance from the root to the tips. As the backbone for our simulations, we used the primate consensus tree (Fig 7, S1 File) from 10kTrees [50], which is the same phylogeny used in recent applications of the IE method [6,10,11]. For each simulated trait, we used the IE algorithm to compute ancestral states, *R*-values, and branch-specific changes measured as standardized directional contrasts (i.e., the value of the descendant minus the ancestor, divided by the square root of the branch length). Although Smaers and Vinicius [4] use *R-*values to represent branch-specific “rates of change”, we have detailed above why we do not consider *R-*values to be rates; thus, in addition to *R*-values, we computed standardized directional contrasts to provide a more direct comparison to the alternative methods discussed below.

The R package adephylo [51] provided functions for manipulating phylogenies that were utilized in coding the IE algorithm. When this project began, no published code for the IE algorithm was available, leading us to write our own script for implementing the method. R code has since been made publically available by the original author [52]. Comparisons revealed our code produced identical results to the newly public code. Given that our code was integrated with other functions written to carry out the present study, we chose to run all analyses with our code and have provided our IE script as supporting information (S2 File). R code for reproducing our entire simulation study and associated figures is available on GitHub [53].

For comparison to IE, we implemented a version of directional contrasts we term Partially Independent Directional Contrasts (PIDC) with ancestral states computed using PIC. The PIC algorithm computes *n*– 1 statistically independent ancestral state values at internal nodes, where the value at each internal node represents the local maximum likelihood estimate of the ancestral state under a BM model of evolution [54]. Once ancestral states were estimated, we computed standardized directional contrasts as described above for IE. Because PIC assumes there is no correlation between trait values and their variance, data were log-transformed prior to analysis. We implemented PIC with the ace function in the ape package [55]. R code for PIDC is also available as supporting information (S3 File).

We examined the means and distributions of estimated ancestral states and branch-specific changes for IE and PIDC. In order to make estimates comparable between IE and PIDC, we log-transformed IE ancestral state reconstructions so that all comparisons are made in log-space. We expected PIDC ancestral states and branch-specific changes to be normally distributed around a mean of 0. For the IE algorithm, we expected that the failure to log-transform values prior to computing the adaptive peak (the “upward pull” property described above) would bias ancestral states deeper in the tree towards increasingly large positive values. Accordingly, we expected IE-estimated branch-specific changes to have a negative bias since trait values tend to decrease from the root to the tips of the tree (i.e., ancestors generally have larger trait values than descendants).

We also investigated the distribution of *R*-values across the tree and compared *R*-values on individual branches to the known branch-specific rates of change from the simulated data. If *R*-values are valid estimates of branch-specific rates of change, then there should be a close relationship between estimated *R*-values and simulated changes along individual branches. Given the multiple problematic steps involved in computing *R*-values, we did not expect to see a strong relationship between *R*-values and the simulated data.

### Simulations: correlated evolution of a pair of traits

We simulated the evolution of 500 pairs of traits on the primate phylogeny using the sim.corrs function in the phytools package [49]. We incremented the evolutionary regression coefficient from 0 to 1 across the 500 pairs of traits. We then computed IE branch-specific changes (using both standardized directional contrasts and *R*-values) independently for each trait and estimated the regression coefficient for pairs of IE contrasts. For comparison, we also analyzed pairs of correlated traits with both traditional PIC and PIDC. For PIC, we computed *n*– 1 independent contrasts for each trait and then estimated regression coefficients for each pair of independent contrasts. For PIDC, we computed 2*n*—2 standardized directional contrasts as described in our analysis of individual traits, and then performed regressions for pairs of directional contrasts. For all regression models, we used ordinary least squares fit through the origin.

We examined the relationship between the simulated evolutionary regression coefficients and the estimated regression coefficients for PIC, PIDC, and IE. We predicted that the regression slopes for pairs of IE branch-specific changes would be strongly correlated with the underlying evolutionary regression coefficients for those traits. If this expectation is borne out, it would contradict the claim that the slope of the regression line for a pair of IE branch-specific changes reflects something different than the underlying scaling relationship between the two traits [6]. We expected the PIC and PIDC regression slopes to provide unbiased estimates of the actual evolutionary regression coefficient, and included these results for comparison with IE.

## Results

### Simulations: evolution of a single trait

**Fig 8:**
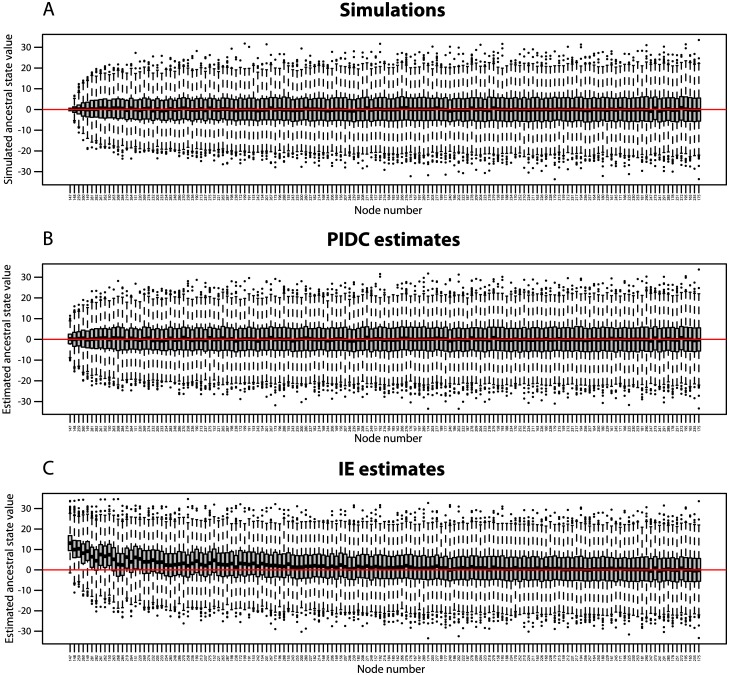
Simulated and estimated ancestral states. Box and whisker plots for (A) simulations of ancestral states, (B) PIDC ancestral state reconstructions, and (C) IE ancestral state reconstructions at each node. Nodes are shown in increasing order based on their distance from the root of the tree. If estimated ancestral states are unbiased, they should be centered on 0. PIDC ancestral state estimates are unbiased (B), while IE ancestral state estimates are biased toward positive values near the root of the tree (C).

As expected, the simulated ancestral states are distributed evenly around 0, with increasing variance from the root to the tips of the tree (Fig 8a). PIDC ancestral state reconstructions show the same pattern (Fig 8b), which is consistent with our expectation that PIDC generates unbiased estimates of ancestral states. In contrast, IE ancestral state reconstructions are strongly biased towards positive values near the root of the tree, and this bias gradually disappears towards the tips of the tree (Fig 8c). The IE estimates at the root of the tree are so strongly biased that the full range of estimates across 1000 simulated data sets barely includes the actual ancestral state of 0.

**Fig 9:**
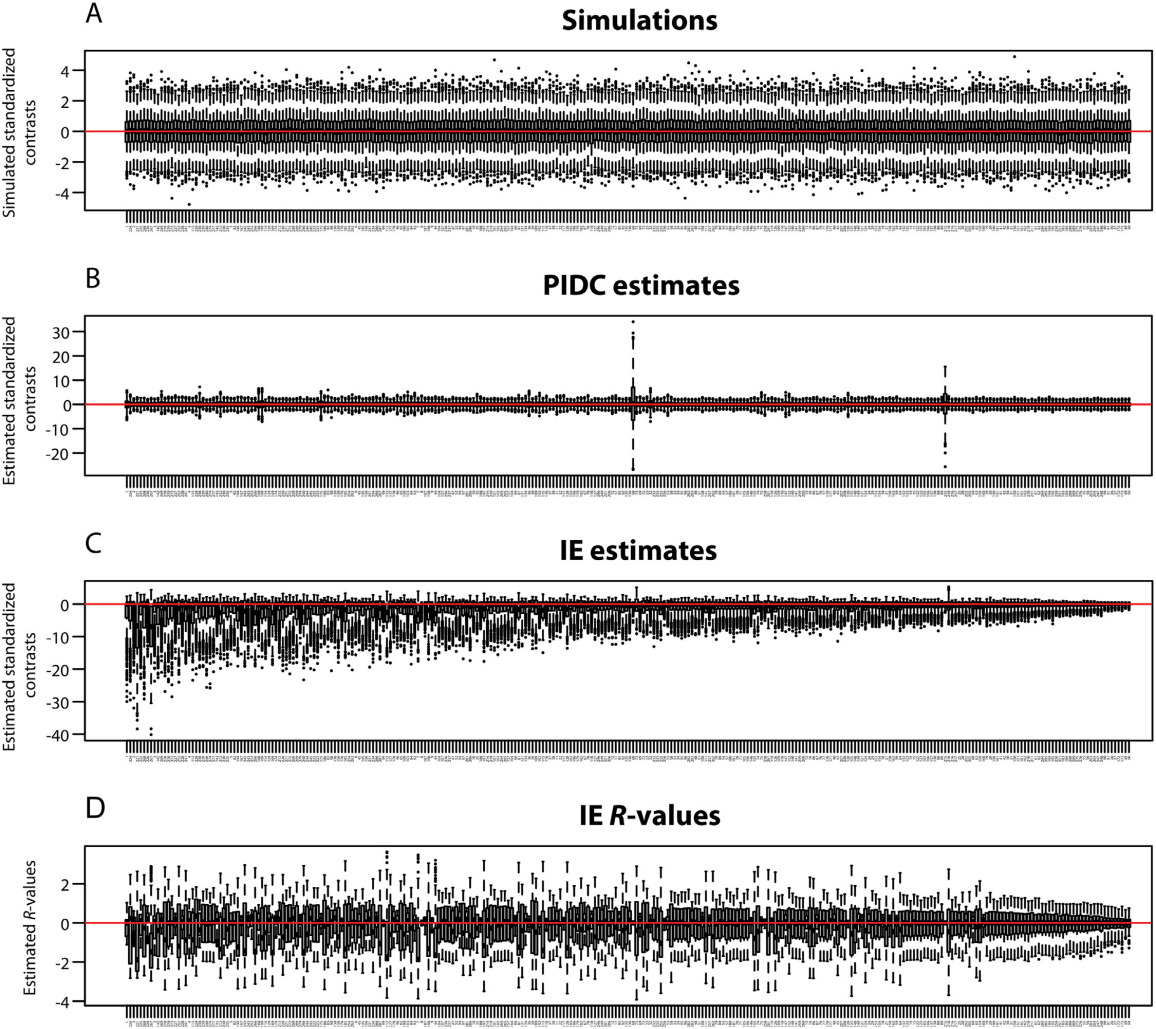
Simulated and estimated branch-specific changes. Box and whisker plots for (A) simulated changes on individual branches, (B) PIDC standardized contrasts on individual branches, (C) IE standardized contrasts on individual branches, and (D) IE *R*-values on individual branches. Branches are shown in increasing order based on their distance from the root of the tree. Branch numbers correspond to the branch labels in Fig 7. Both simulated changes (A) and PIDC estimates (B) are centered on 0, while IE standardized contrasts (C) are biased toward large negative values near the root of the tree. IE *R*-values (D) also have a negative bias, though not as pronounced as for IE standardized contrasts. Larger variances of PIDC estimates are found on shorter branches (B), e.g., the two largest variances occur on branches 68 and 215, which both have branch lengths of 0.01. Variance of both IE standardized contrasts and *R*-values decreases from the root to the tips of the tree (C, D).

The bias in IE ancestral state reconstructions is also readily apparent in the estimated branch-specific changes across the tree. While simulated and PIDC estimated branch-specific changes are evenly distributed around 0 (Fig 9a and 9b), IE estimates based on standardized directional contrasts are strongly skewed towards negative values, particularly near the root of the tree (Fig 9c). This bias is so severe that IE estimates near the root are sometimes mis-estimated by orders of magnitude. We also observe uneven distributions of IE estimated branch-specific changes, with many negative outliers and very few positive outliers (Fig 9c). This reflects the fact that distributions are skewed in the negative direction, because large positive estimates are extremely unlikely relative to large negative estimates. On average, inferred changes near the root of the tree tend to be much larger than changes near the tips, so that the rate of evolution appears to decrease through time.

**Fig 10:**
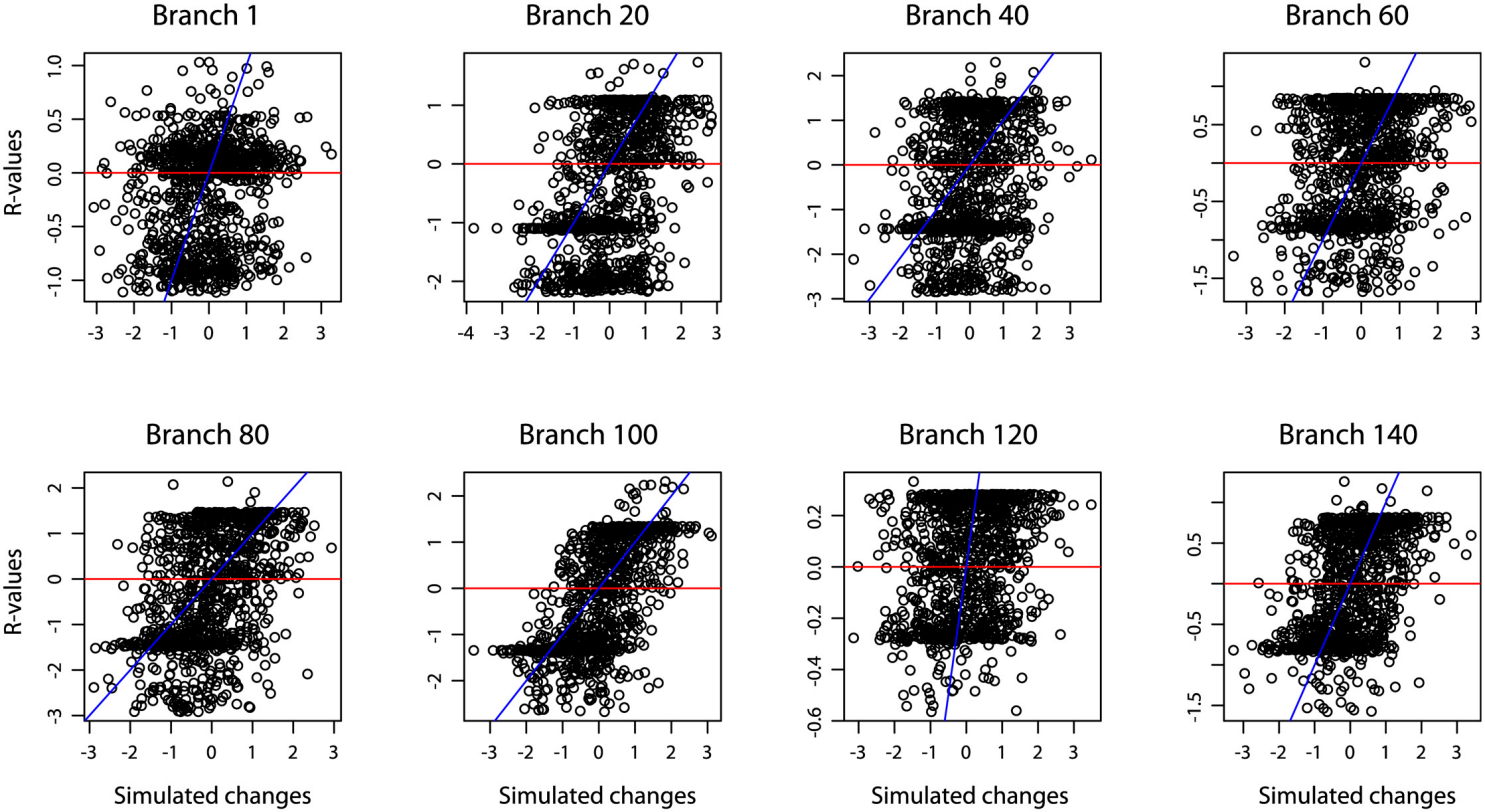
*R*-values and simulated branch-specific changes. Scatterplots of *R*-values against simulated branch-specific changes for representative branches of the phylogeny. Branch numbers correspond to branch labels in Fig 7. If *R*-values provide accurate estimates of branch-specific changes, then they should fall along the line *y* = *x* (drawn in blue) and be evenly distributed above and below the line *y* = 0 (drawn in red). The slight negative bias in *R-*values can be seen in the higher concentration of points below the red line, while the unevenly and widely scattered points about the blue line show that *R*-values behave erratically and provide poor estimates of the actual evolutionary change along branches.

Our comparison of *R*-values to actual branch-specific changes reveals that *R*-values behave erratically and provide very poor estimates of branch-specific change. The variance of *R*-values decreases towards the tips of the tree and shows a slight negative bias (Fig 9d). Although a general positive trend appears to exist between *R*-values and simulated branch-specific changes, there is an enormous amount of scatter in the data, and we observed highly irregular distributions of *R*-values (Fig 10).

### Simulations: correlated evolution of a pair of traits

**Fig 11:**
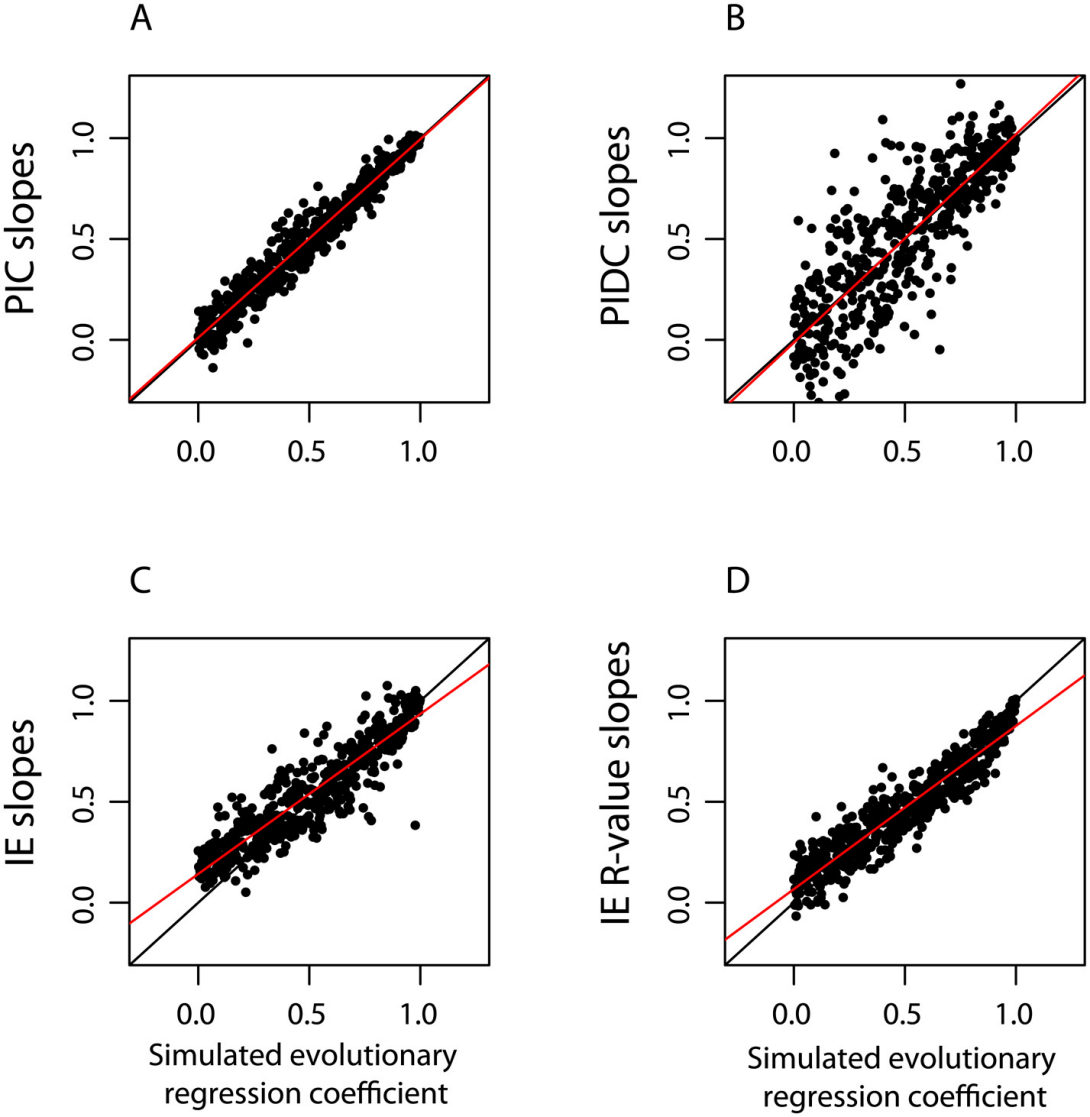
Relationship between simulated evolutionary regression coefficients and estimated slopes. Estimated evolutionary regression coefficients were regressed against simulated evolutionary regression coefficients for A) PIC, B) PIDC, C) IE standardized directional contrasts, and D) IE *R*-values. If estimates are unbiased, points should fall along the line *y* = *x*, shown in black. The observed regression lines for each type of estimate are shown in red. PIC and PIDC produce unbiased estimates of the evolutionary regression coefficient (A and B). In contrast, while IE estimates are correlated with the underlying evolutionary regression coefficent, IE produces overestimates when the true evolutionary regression coefficient is low to moderate, and underestimates when the evolutionary regression coefficient is high (C, D).

We found a close linear relationship between the simulated regression coefficients and the estimated regression coefficients for all three of the methods: PIC, PIDC, and IE. Thus, our simulations provide strong support for our prediction that regressing IE branch-specific changes for two traits captures the evolutionary regression coefficient or allometric scaling relationship for those traits, but does not provide novel information about relative historical rates of evolution for the two traits (contrary to interpretations of [6]). The results for PIC and PIDC confirm Pagel’s [40] findings that whether cross-sectional contrasts (i.e., comparisons between sister taxa, as in PIC) or directional contrasts (i.e., comparisons between ancestors and descendants, as in PIDC and IE) are used in regression analyses, the same parameter is estimated. Regressing estimated slopes against simulated slopes reveals that both PIC (*β* = 0.98; Fig 11a) and PIDC (*β* = 1.03; Fig 11b) provide unbiased estimates, while IE provides the least accurate estimates whether standardized direction contrasts (*β* = 0.78; Fig 11c) or *R*-values were used (*β* = 0.81; Fig 11d).

## Discussion

We used computer simulations to demonstrate that the IE method produces severely biased estimates of ancestral states and branch-specific changes when data are simulated under a BM model. Specifically, the IE method systematically overestimates ancestral states and underestimates branch-specific changes, while inferring excessively large changes near the root of the tree. These trends are less extreme for *R*-values, however comparison of *R*-values to simulated changes along individual branches reveals they have a tenuous relationship to the simulated branch-specific changes, and are mostly random noise. Thus, our findings reject the proposal that the IE method produces equivalent results to a BM model when the assumptions of BM are met [4]. The implicit model realized by the IE algorithm can be described as “evolution with a decrease in both the mean and variance of the trait over time.” While this model might be appropriate in some cases, it is certainly not a general expectation of the evolutionary process; importantly, the IE method has no way of assessing whether its implicit model fits a given data set better than alternatives. Additionally, we have shown that the slope of the regression of IE branch-specific rates of change for a pair of traits is a linear function of the evolutionary regression coefficient between those traits, indicating that the IE regression slope estimates the same parameter as the phylogenetic regressions used in studies of allometry.

It appears that the development and subsequent applications of IE are characterized by misunderstandings of log-transformation, allometry, and existing PCMs. Despite the well-known and broadly accepted approach of log-transforming geometrically normal data [1,14,16–19], the IE method uses a distance metric that fails to measure proportional change accurately over biologically reasonable ranges (Fig 2). Smaers and Vinicius’ [4] claim that “gradual models of evolution” do not account for proportional change, but this is not true: BM models assume that there is no correlation between trait means and their variance, thus if the raw data exhibit geometric normality, log-transformation is a standard procedure to remove the correlation. The failure to log-transform geometrically normal data likely accounts for the poor performance of alternative PCMs in Smaers and Vinicius’ [4] simulations. We have shown that the combination of inadequate treatment of geometrically normal data and a biased distance metric generates the discrepancy between the “ancestral state” and “adaptive peak” in the IE algorithm (Fig 4; Appendix 1), and when the data are log-transformed and distances are computed as the difference between logged values, the “adaptive peak” disappears. While the general concept of incorporating selection towards adaptive peaks into models of evolution is valid and interesting, the IE method fails to provide a theoretically grounded approach that achieves this goal.

### Reinterpretation of past studies that use the IE method

The IE method has been been applied in three ways: to examine variation in the rates of change for a single trait across the branches of a phylogeny [6–11], to compare the coevolution of two traits by examining the relationship between the branch-specific rates of change for a pair of traits [6–9], and to test macroevolutionary hypotheses of evolutionary rates [12,13]. The arguments advanced in these papers should be reevaluated to the extent that they depend on results of the IE method.

The majority of studies that have utilized the IE algorithm have only depicted the results with graphic representations [6–13], though there is a truncated example table in the supplementary material of Smaers et al. ([6], their Table S2). This makes it difficult to directly assess the systematic positive bias of ancestral trait values and negative bias of *R-*values that we recovered in our simulations. However, the distributions of points in Fig 3 of Smaers et al. [6], as well as Figs 4 and 5 of Smaers et al. [7], likely reflect the patterns we have described: the presence of many large negative “rates” and few positive “rates” is consistent with the bias toward negative *R-*values in our simulations, and the clustering of small “rates” near the center of each plot is consistent with our observation that *R-*values tend to be small on the numerous branches near the tips of the tree (Figs 9d and 10). The highly positively skewed distribution of evolutionary “rates” reported by Goswami et al. [12] is also consistent with our results.

For studies which have utilized the IE method to investigate the evolution a single trait [6–11], the results have been underwhelming despite the purported power of the method. For example, Smaers and Soligo [9] conclude, “Different neocortical and cerebellar areas contribute differently to explaining different mosaic patterns in different lineages” (p. 6). This conclusion is undoubtedly true, but it provides no insights that could not be gleaned from the observed variation in the tips of the phylogeny. These studies primarily focus on *R*-values, which are interpreted as evolutionary rates of change. However, as we have shown, *R*-values are poor estimators of change over time (Figs 9 and 10), so that the evolutionary scenarios described in these studies are likely to be spurious.

Studies that have utilized the IE method to examine the correlated evolution of two traits have approximated the allometric scaling relationship of those two traits [6–9]. For instance, Smaers et al. [6] demonstrate that bats, carnivores, and primates exhibit different interspecific allometric relationships between brain mass and body mass, which is already revealed by the PGLS slopes in their Fig 1. The IE method provides no information that is not apparent in the variation observed across the tips of the phylogeny: it is clear that body mass varies more than brain mass among extant bats, carnivorans, and primates. Assuming that each of these orders diversified from common ancestors, we should expect body mass rates of change to be greater than brain mass rates of change. The important biological questions to ask are how, why, and when different allometric relationships emerged among mammalian orders. As we have seen, the IE method cannot meaningfully address these questions. Indeed, relative to existing PCMs, the IE method does a poor job of estimating the allometric relationship between two variables (Fig 11).

Two recent studies have used IE to test broad macroevolutionary hypotheses regarding evolutionary rates. Goswami et al. [12] use IE-inferred rates of change to test the hypothesis that strong integration in cranial modules is associated with slower rates of evolution, and conclude, “Perhaps surprisingly, our analyses did not support a significant correlation” (p. 10). Most recently, Jones et al. [13] use *R-*values to test the hypothesis of an adaptive radiation during the terrestrial-aquatic transition within Carnivora, and because *R*-values were not large at the base of Pinnipedia, the authors conclude there is no evidence for an adaptive radiation. Given the tenuous relationship between *R*-values and actual rates of change (Fig 10), IE-based analyses are poor tests of these macroevolutionary hypotheses.

### Alternative approaches to modeling adaptive peaks and variable rates of evolution

Smaers and Vinicius [4] are not the first researchers to consider selection toward adaptive optima or heterogeneity in evolutionary rates in PCMs. A large body of research has focused on formally modeling evolution towards adaptive optima by drawing upon the Ornstein-Uhlenbeck (OU) model of evolution [56]. In an OU model, traits evolve under a BM model with variance σ^2^, but are also pulled toward an adaptive optimum *θ* with strength of selection *α*, such that evolutionary change in trait *Y* over time *t* is described by

$$dY_{t}=-\alpha \left(Y_{t}-\theta \right)dt+\sigma dB_{t}$$ (4)

where *dB*_*t*_ represents a Brownian process (i.e., random change drawn from a normal distribution with mean 0 and variance 1) and *σ* is a multiplier for the variance of the Brownian process. When *α* = 0, this corresponds to a model with no selection strength, thus the first term drops out of the equation to leave a simple Brownian motion model. When *α* is large, this yields a model in which trait evolution is strongly biased toward the adaptive optima *θ*. The parameter *α* is sometimes referred to as a “rubber band” parameter, because the further a trait wanders from the adaptive optima, the more strongly it is pulled back. Together, the parameters *σ*, *α*, and *θ* define the selection regime for the trait.

The OU model has been used to model heterogeneity across phylogenies in the adaptive optima [57] and in the strength of selection [58]. The adaptive optima itself can also be modeled with a Brownian motion process [59]. Many of these methods depend on specifying *a priori* positions for shifts in the selection regime on the phylogeny and then comparing the more complex model to a simpler model to determine whether there is statistical support for multiple selection regimes across the phylogeny. More recent methods have focused on identifying shifts in the selection regime without *a priori* specification. For instance, Thomas and Freckleton [60] and Ingram and Mahler [61] have proposed stepwise model selection procedures for identifying shifts in the selection regime anywhere in the phylogeny, while Eastman et al. [62], Rabosky [63], and Uyeda and Harmon [64] have developed Bayesian approaches to identifying selection regime shifts without *a priori* hypotheses. All of these methods involve explicit statistical models with careful attention to bias and error in parameter estimation, and they all incorporate mechanisms to limit the number of parameters in order to find the optimal trade-off between model complexity and goodness-of-fit. Their development and evaluation continues to be an active and fruitful area of research, and challenges associated with fitting these models and using them for statistical inference are increasingly being appreciated [65,66].

## Conclusions

Over the past several decades, PCMs have increasingly become an integral part of evolutionary biology. Unfortunately, methodological papers are often riddled with jargon and mathematics that render them opaque to evolutionary biologists who are interested in using PCMs. Moreover, method developers are often not involved in the publication or peer review of biological research that utilizes PCMs. The disconnect between developers and users creates a wide space for the misuse of PCMs in evolutionary biology. Researchers should be wary of unfamiliar methods that are not widely used, are not implemented in published software packages, and have not been tested with extensive simulation studies. In the case of the IE method, code was not published simultaneously with the method’s original description, the method has not been used outside of collaborations with the primary developer, and the method has not been thoroughly evaluated by independent parties. Studies using the method have been vague on how the method works, frequently present only graphic representation (but not quantitative data) of the method’s results, and refer readers only to the original paper and its limited set of simulations.

To our knowledge, this is the first study to independently evaluate the properties of the IE method developed by Smaers and Vinicius [4]. We have identified eight problems with the IE method and its application. First, the IE distance metric is biased and generates undefined values. When we used an unbiased distance metric, the adaptive peak and ancestral state values are identical (Fig 4); this equivalence will always be true when branch lengths are equal and the value of the “peak” is between the two tip values (Appendix 1). Second, by imbedding a data transformation step into the method, the IE method assumes all data have similar error structures, which is not true. Third, the IE method utilizes Wagner tree formulas in a geometric context, but these formulas are not appropriate for calculating distances from a triangle vertex to the centroid. Fourth, while *R*-values have been described and analyzed as “rates of change” [4,6–13], these values are not true evolutionary rates. The simulations of this study reveal that *R*-values are erratic and do not have a meaningful relationship with actual rates of change between ancestors and descendants. Fifth, since geometrically normal data are transformed after the adaptive peak is calculated, large values cause the adaptive peak and ancestral state values to be overestimated. Simulations show this is particularly problematic at the base of the phylogeny, where values can be overestimated by an order of magnitude or more. Sixth, similar to another directional contrast method (Minimum Evolution), the IE method estimates 2*n* − 2 parameters from *n* observations. This extensive pseudoreplication is antithetical to a central goal of phylogenetic comparative methods: to control for the statistical non-independence of species data. Seventh, there is no compelling theoretical reason for extending Felsenstein’s [20] independent contrast formula to the entire phylogeny to calculate “adaptive peaks”. Finally, analyses that examine the relationship between branch-specific rates of change for two traits do not reveal the selective pressures on one trait over the other; rather, they provide a biased estimate of the “evolutionary regression coefficient”, or the scaling relationship between the two variables. Traditional PCMs such as phylogenetically independent contrasts [20] provide unbiased estimates of this coefficient.

Given these problems, we believe we have shown that the Independent Evolution method is not a viable phylogenetic comparative method. However, we have tried to highlight alternative PCMs with similar goals that have strong theoretical foundations and validation from simulation studies (although these methods are not without limitation). Due to the profound theoretical weaknesses of the IE method, we see no way for the method to be salvaged with subsequent revisions.

## Appendix 1 –Algebraic proof of equivalence of adaptive peak and ancestral state values

The adaptive peak and ancestral state values are equivalent (*A*_1,2_ = *AP*) when the difference between logged values is used as the distance metric, branch lengths are equal (*b*_1_ = *b*_2_), and *AP* lies between *x*_1_ and *x*_2_. Here we consider only the condition *x*_1_ < *AP* < *x*_*2*_; by symmetry the proof is also true when *x*_1_ > *AP* > *x*_2_.

Given:

By Fig 5.5, when *AP* < *x*_2_:

$$s_{2}=x_{2}-AP\Rightarrow x_{2}=AP+s_{2}$$ (5)

By Fig 5.5, when *AP* > *x*_1_:

$$s_{3}=AP-x_{1}\Rightarrow x_{1}=AP-s_{3}$$ (6)

By rearranging Eq 5 and Eq 6, and substituting:

$$AP=x_{1}+s_{3}=x_{2}-s_{2}\Rightarrow s_{3}+s_{2}=x_{2}-x_{1}$$ (7)

By Fig 5.8, where *R*_1_ = *s*_3_ and *R*_2_ = *s*_2_ when *b*_1_ = *b*_2_:

$$A_{1,2}=\frac{\frac{x_{1}}{R_{1}}+\frac{x_{2}}{R_{2}}}{\frac{1}{R_{1}}+\frac{1}{R_{2}}}=\frac{\frac{x_{1}}{s_{3}}+\frac{x_{2}}{s_{2}}}{\frac{1}{s_{3}}+\frac{1}{s_{2}}}=\frac{s_{3}x_{2}+s_{2}x_{1}}{s_{3}+s_{2}}$$ (8)

By substituting Eq 7 into the denominator of Eq 8, and substituting Eqs 5 and 6 into the numerator of Eq 8, we can prove algebraically that *A*_1,2_ = *AP*:

$$A_{1,2}=\frac{s_{3}x_{2}+s_{2}x_{1}}{s_{3}+s_{2}}=\frac{s_{3}x_{2}+s_{2}x_{1}}{x_{2}-x_{1}}=\frac{s_{3}\left(AP+s_{2}\right)+s_{2}\left(AP-s_{3}\right)}{x_{2}-x_{1}}=\frac{s_{3}AP+s_{3}s_{2}+s_{2}AP-s_{3}s_{2}}{x_{2}-x_{1}}=\frac{s_{3}AP+s_{2}AP}{x_{2}-x_{1}}=\frac{AP\left(s_{3}+s_{2}\right)}{x_{2}-x_{1}}=\frac{AP\left(x_{2}-x_{1}\right)}{x_{2}-x_{1}}=AP$$ (9)

## Supporting Information

S1 File Nexus file of primate phylogeny used for simulations. (NEX)

S2 File R code for IE algorithm. (R)

S3 File R code for PIDC algorithm. (R)
